# A Novel Gas Sensor Based on MgSb_2_O_6_ Nanorods to Indicate Variations in Carbon Monoxide and Propane Concentrations

**DOI:** 10.3390/s16020177

**Published:** 2016-01-30

**Authors:** Héctor Guillén-Bonilla, Martín Flores-Martínez, Verónica-María Rodríguez-Betancourtt, Alex Guillen-Bonilla, Juan Reyes-Gómez, Lorenzo Gildo-Ortiz, María de la Luz Olvera Amador, Jaime Santoyo-Salazar

**Affiliations:** 1 Departamento de Ingeniería de Proyectos, CUCEI, Universidad de Guadalajara, 44410 Guadalajara, JAL, Mexico; doctorado.materiales@cucei.udg.mx; 2 Departamento de Químicas, CUCEI, Universidad de Guadalajara, 44410 Guadalajara, JAL, Mexico; veronica.rodriguez@red.cucei.udg.mx; 3 Departamento de Ciencias Computacionales e Ingenierías, CUVALLES, Universidad de Guadalajara, Carretera Guadalajara-Ameca Km 45.5, 46600 Ameca, JAL, Mexico; alexguillenbonilla@gmail.com; 4 Facultad de Ciencias, Universidad de Colima, 28045 Colima, COL, Mexico; reyesgj@ucol.mx (J.R.-G.); lorenzo.gildo@gmail.com (L.G.-O.); 5 Nanociencias y Nanotecnología, Centro de Investigación y de Estudios Avanzados del Instituto Politécnico Nacional, 07360 México, DF, Mexico; 6 Departamento de Ingeniería Eléctrica-SEES, Centro de Investigación y de Estudios Avanzados del Instituto Politécnico Nacional, 07360 México, DF, Mexico; molvera@cinvestav.mx; 7 Departamento de Física, Centro de Investigación y de Estudios Avanzados del Instituto Politécnico Nacional, 07360 México, DF, Mexico; jsantoyo@fis.cinvestav.mx

**Keywords:** nanorods, trirutile, sensitivity, carbon monoxide, propane

## Abstract

Bystromite (MgSb_2_O_6_) nanorods were prepared using a colloidal method in the presence of ethylenediamine, after a calcination step at 800 °C in static air. From X-ray powder diffraction analyses, a trirutile-type structure with lattice parameters *a* = 4.64 Å and *c* = 9.25 Å and space group *P*4_2_/*mnm* was identified. Using scanning electron microscopy (SEM), microrods with sizes from 0.2 to 1.6 μm were observed. Transmission electron microscopy (TEM) analyses revealed that the nanorods had a length of ~86 nm and a diameter ~23.8 nm. The gas-sensing properties of these nanostructures were tested using pellets elaborated with powders of the MgSb_2_O_6_ oxide (calcined at 800 °C) at temperatures 23, 150, 200, 250 and 300 °C. The pellets were exposed to different concentrations of carbon monoxide (CO) and propane (C_3_H_8_) at these temperatures. The results showed that the MgSb_2_O_6_ nanorods possess excellent stability and high sensitivity in these atmospheres.

## 1. Introduction

The global scientific community has recently come to understand the importance of better control over the high-polluting gas emissions sent into the atmosphere by motor vehicles and industries, mainly in big cities [1]. The results are ecological instability and global warming. These gas emissions include CO, CO_2_, NO_2_, NO and SO_2_ [2,3], which have triggered health problems connected with respiratory diseases among dense populations. In order to avoid these conditions and to protect the environment, it is important to know the concentrations of these gases on an ongoing basis, by constantly monitoring strategic points. Therefore, extensive research has been conducted into the field of gas sensors. One of the main lines of research is based on the preparation of inorganic materials like oxide semiconductors, which are chemically stable and capable of operating in a wide range of temperatures. The most widely studied semiconductors for this application include LaFeO_3_, SnO_2_, ZnO and WO_3_ compounds, among others [4,5,6]. However, during the past few years, oxides with a trirutile-type structure have also aroused interest for this purpose because these materials exhibit good electrical response, as well as short recovery and response times [7]. In order to investigate their gas sensing properties, P. T. Moseley *et al.* [8] prepared transition metal tantalates with trirutile-type structure (CoTa_2_O_6_ and NiTa_2_O_6_). They found that this compound group exhibits changes in electrical conductivity, which can be used to elaborate reliable gas detectors. Tamaki *et al.* [9] synthesized the trirutile-type ZnSb_2_O_6_, finding good sensitivity at 300 °C in an atmosphere of H_2_S (0.01 ppm). The good sensitivity of this material could be in part attributed to the porous structure of the film made of ZnSb_2_O_6_.

With regard to advances in the preparation of these inorganic materials, different methods have been used in order to obtain materials with nanometric particle sizes (10^−9^ m) and improved microstructural characteristics. Among the most commonly used methods are non-aqueous, solution-polymerization, aerosol and colloidal routes [10,11,12,13,14]. The colloidal method is more used nowadays because this process produces materials with unique morphologies and very small particle sizes (<100 nm) [15]. Matijevic *et al.* [16] have synthesized inorganic materials based on the colloidal method (oxides and sulfides among others). Basically, when colloidal particles are precipitated inside homogeneous dispersions, diverse morphologies can be obtained. Libert *et al.* [17] reported the formation of microspheres using a similar procedure. In this work, MgSb_2_O_6_ (magnesium antimony oxide, also known as Bystromite) nanorods were synthesized by a colloidal method for gas sensing purposes. The characterization of the MgSb_2_O_6_ powders was made by scanning and transmission electron microscopy (SEM and TEM, respectively). In addition, different sensitivity tests were performed on pellets of MgSb_2_O_6_, which showed high sensitivity in carbon monoxide (CO) and propane (C_3_H_8_) atmospheres at relatively low temperatures.

## 2. Experimental Section

### 2.1. Synthesis of MgSb_2_O_6_ Nanorods

The synthesis of MgSb_2_O_6_ nanorods was performed at room temperature by a colloidal method [7,15,16]. In a typical synthesis, 1.28 g (0.005 mol) of Mg(NO_3_)_2_·6H_2_O (Mallinckrodt, Dublin, Ireland ), 2.28 g (0.01 mol) of SbCl_3_ (Sigma-Aldrich, St. Louis, MO, USA), and 4 mL of ethylenediamine (Sigma, St. Louis, MO, USA) were used. The reagents were dissolved separately in 5 mL of ethyl alcohol (Golden Bell, Anaheim, CA, USA), except for the ethylenediamine, which was dissolved in 10 mL of the same solvent. The three solutions showed transparency and were stirred for one hour. Following that, the solutions based on ethylenediamine and magnesium nitrate were mixed, obtaining a white solution and the formation of a large coagulum. Afterwards, a solution based on antimony chloride was slowly added, generating a white refined precipitate. The resulting solution (or colloidal dispersion) was kept under stirring for 24 h and the solvent was then evaporated through microwave radiation. The exposure of the colloidal solution to the radiation was done in short periods of time of 20 to 30 s, reaching its maximum temperature of 70 °C. The power applied for the evaporation of the solvent was of 178 W, using a home microwave device (General Electric model JES769WK, Louisville, KY, USA). The energy absorbed by the colloidal solution was calculated to be 45 kJ. The obtained precursor material was a white paste, and it was dried at 200 °C for 8 h. Afterwards, the obtained powders were calcined from 800 °C and up at a rate of 100 °C/h, yielding white powders. The calcinations were carried out in a Vulcan 3-550 oven (DENTSPLY NeyTech Division, Yucaipa, CA, USA), which had a programmable temperature control.

### 2.2. Physical Characterization of MgSb_2_O_6_ Powders

The crystalline structure of the MgSb_2_O_6_ (calcined at 800 °C) was analyzed by X-ray powders diffraction at room temperature, using a D500 Siemens diffractometer (Siemens, Munich, Germany) with a Cu-Kα radiation (λ = 0.1518 nm). The 2θ scanning range was from 10° to 70° with a size step of 0.02° and a time step of 1 s. The morphology of the MgSb_2_O_6_ powders was characterized by means of a scanning electron microscopy system (JEOL JSM-6390LV; Jeol, Inc., Dearborn, MI, USA) in high vacuum and using the secondary electron emission. Size and shape of the nanorods were analyzed with a transmission electron microscopy (TEM) system (Jeol, JEM-2010; Jeol, Inc., Boston, MA, USA) with a B_6_La filament at 200 kV. For the TEM analysis, the powders were previously dispersed for 5 min in isopropyl alcohol and supported in a formvar/carbon grid on copper 400 mesh.

### 2.3. Pellets Preparation for Gas Sensitivity Analysis

**Figure 1:**
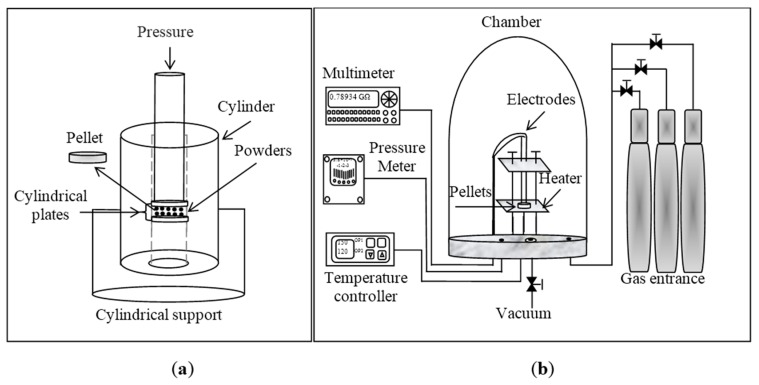
(**a**) Diagram of the device used to compact the powders of MgSb_2_O_6_ oxide; (**b**) arrangement used for the sensitivity testing in controlled atmospheres and temperatures [18].

The sensing properties were analyzed using MgSb_2_O_6_ pellets. To elaborate the pellets, 0.4 g of MgSb_2_O_6_ powders were pressed at 20 ton during 160 min with a manual pressing machine (Simplex Ital Equip–25 tons (México, Mexico), see Figure 1a. The obtained pellets had a diameter of 12 mm and a thickness of 0.5 mm. The sensitivity testings were done inside a measurement vacuum chamber with 10^−3^ torr of capacity. Gas concentration and partial pressure were controlled using a TM20 Leybold detector (Oerlikon Leybold Vacuum, Cologne, Germany). Electric resistance measurements were carried out by means of a digital multimeter (model Keithley 2001; Keithley Instruments, Inc, Cleveland, OH, USA). A diagram of the equipment is shown in Figure 1b. This sensing system was tested in our previous work [18].

## 3. Results and Discussion

### 3.1. XRD Analysis

**Figure 2:**
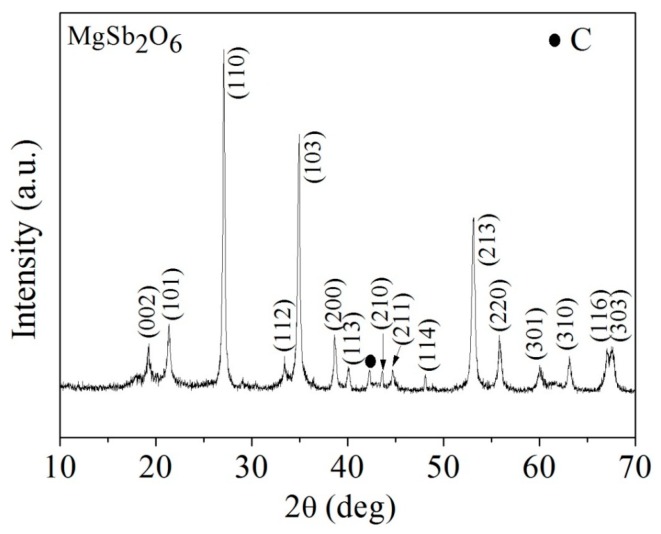
X-ray diffraction pattern of MgSb_2_O_6_ powders calcined at 800 °C and prepared by the colloidal method.

Figure 2 shows a typical diffractogram of the MgSb_2_O_6_ powders after the thermal treatment at 800 °C. This result reveals the presence of the main phase corresponding to MgSb_2_O_6_, which was identified by the file JCPDF No 88-1725. According to this, the MgSb_2_O_6_ is a trirutile-type oxide (showing tetragonal structure) with cell parameters *a* = 4.64 Å and *c* = 9.25 Å, and space group *P*4_2_/*mnm* [19]. In addition, the width of the diffraction peaks was an indication of the nanometric size of the particles [7,20]; the presence of slight fluorescence indicated high crystallinity. Furthermore, a secondary phase was identified through the JCPDF No 26-1083 file, localized on the angular position 2θ = 42.1°, which corresponds to carbon (C). The secondary phase in the MgSb_2_O_6_ at 800 °C is attributed to that carbon, which could be produced during the thermal decomposition of organic material when the sample was calcined inside a closed ceramic crucible; this has been discussed in previous works [21]. These results are consistent with those reported in the literature, where the same oxide and similar ones were used [22]. As a comparison, Mizoguchi and Woodward [23] synthesized the MgSb_2_O_6_ based on a wet-chemical method, where the phase was obtained at higher temperature (1047 °C). In the present work, as previously mentioned the oxide was obtained at a lower temperature (800 °C) but employing an alternative synthesis procedure (the microwave-assisted colloidal method).

### 3.2. Scanning Electron Microscopy Analysis

**Figure 3:**
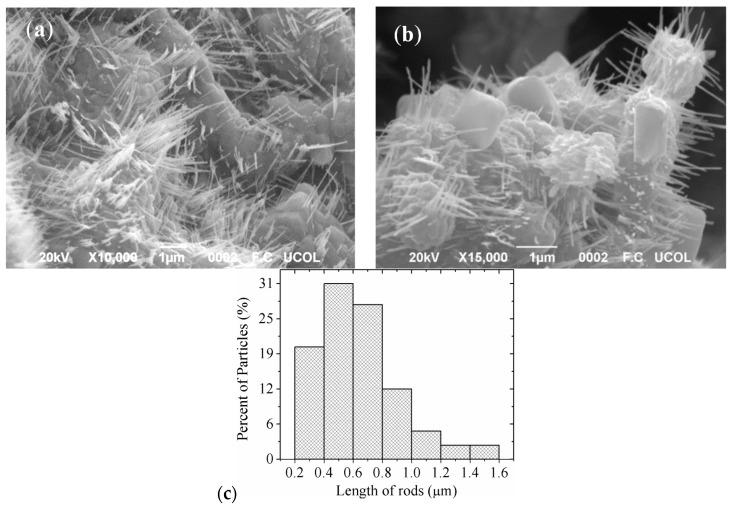
SEM images of MgSb_2_O_6_ powders calcined at 800 °C at different magnifications: (**a**) 10,000×; (**b**) 15,000×; and (**c**) size distribution of the rods.

In order to observe the morphology of the MgSb_2_O_6_ oxide, scanning electron microscopy (SEM) was used. Figure 3 shows two typical SEM images of the oxide’s surface at different magnifications. A great number of rods are observed, which formed over the whole surface. These microstructures have grown individually and in all directions, taking a microplate as a substrate (Figure 3a). It can be seen in Figure 3b that tiny linked crystals forming a polycrystalline surface constitute the microbase. The microrods’ sizes were estimated in the range 0.2–1.6 μm, with an average of ~0.60 μm and a standard deviation of ± ~0.23 μm; see Figure 3c to check these assertions. 

The effect of ethylenediamine in the formation of very small structures of materials, like nanorods and nanowires, has been discussed in previous studies [7,24,25]. The ethylenediamine acts as a template, which is incorporated first into the inorganic framework and then escapes from it during the thermal treatment, forming particles of desired morphologies [25]. In this work, we achieved the growth of rods on the surface of the MgSb_2_O_6_ incorporating the ethylenediamine during the synthesis process. The colloidal-dispersion formation (nucleation and growth) has been established by the LaMer and Dinegar mechanisms [7,15,18,26]. These authors proposed three theoretical principles for this: (1) the concentration of stable reagents in colloidal dispersions increases gradually; (2) the concentration of reagents reaches the limit of oversaturation and nucleation occurs faster, forming the nuclei of crystals; (3) the particles’ growth begins and their morphology is now clearly discernible. The microstructures are attributed to the formation of stable nuclei, which were formed during a strong reaction caused by the ethylenediamine (the colloidal dispersion) [27,28].

### 3.3. Transmission Electron Microscopy Analysis

**Figure 4:**
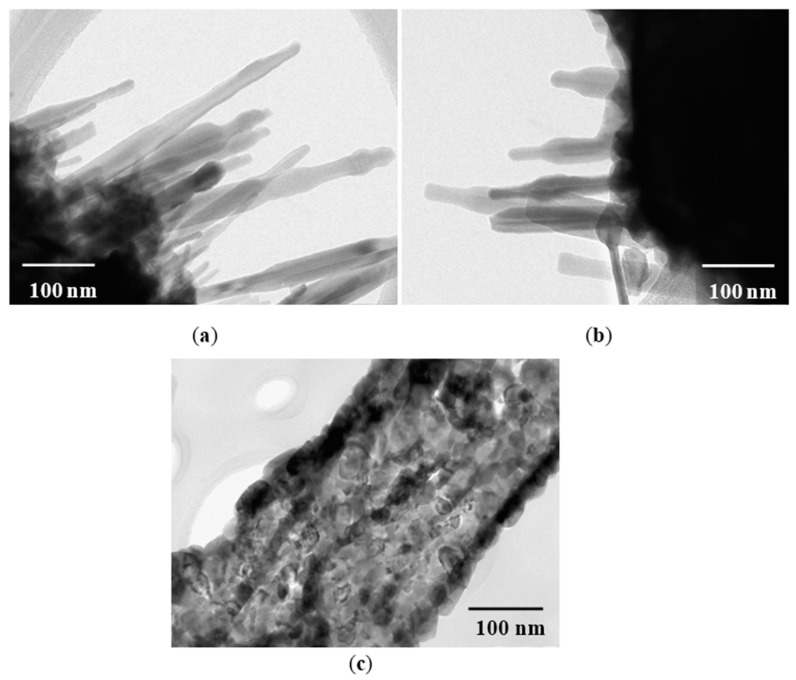
TEM images of MgSb_2_O_6_ powders calcined at 800 °C: (**a**,**b**) nanorods; (**c**) surface of a rod with nanoparticles.

Figure 4 shows three transmission electron microscopy (TEM) images of the MgSb_2_O_6_ morphology in samples obtained at 800 °C. The black zones are caused by the small electron beam transmission through the sample; this effect is caused by the particle agglomeration over the material’s surface. Figure 4 corroborates the morphology observed by Scanning Electron Microscopy (SEM). In Figure 4a,b, the agglomeration of nanorods of different size, sitting on a microbase, is clearly visible. The size of the biggest nanorods was estimated of ~303 nm; the shorter ones had a calculated size of ~86 nm. The surface of a rod can be observed in Figure 4c, where some porosity and the formation of nanoparticles’ clusters distributed over the surface are visible.

**Figure 5:**
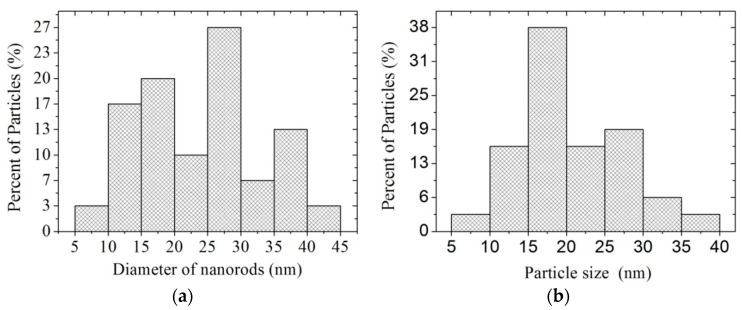
Size distribution of MgSb_2_O_6_: (**a**) nanorods’ diameter; and (**b**) size of nanoparticles.

Figure 5 depicts the size distribution of the nanorods and nanoparticles of the MgSb_2_O_6_ oxide. The estimated nanorod diameter was in the range of 5–45 nm and ~23.8 nm on average, with a standard deviation of ±~10 nm (Figure 5a). The nanoparticles size was estimated in the range of 5–40 nm, ~20 nm on average, and a standard deviation of ±~7 nm (Figure 5b).

**Figure 6:**
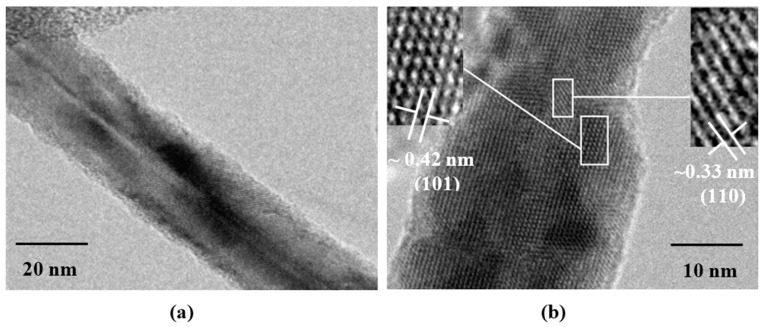
HRTEM Image showing resolved lattice fringes of a nanorod: (**a**) nanorod divides it into 2 sections; (**b**) surface of a nanorod showing one selected zone of the MgSb_2_O_6_.

Figure 6 shows two high-resolution images (HRTEM). Figure 6a depicts one nanorod with a diameter of ~16 nm. In this particle, a fringe located all along the nanorod divides it into 2 sections. The minimum diameter of the nanorod was estimated of ~8 nm. In addition, resolved lattice fringes over the surface of the nanorods were observed, confirming its crystalline nature. This is more evident in Figure 6b. The distance *d* between planes was measured on two different zones, based on intensity profiles. These distances were ~0.42 nm and ~0.33 nm, which respectively correspond to the distance between the planes (101) and (110) in the tetragonal structure of the MgSb_2_O_6_. These planes have maximum diffraction angles at 2θ = 21.35° and 27.16°, which can be observed in the X-ray diffraction pattern (see Figure 1).

### 3.4. Sensing Properties

In order to evaluate the MgSb_2_O_6_ as a potential gas sensor, it was necessary to prepare pellets (~500 μm) of the material, measuring the changes in the electrical resistance at different gas concentrations and operating temperatures. The pellets of MgSb_2_O_6_ were exposed to carbon monoxide (CO) and propane (C_3_H_8_) flows at concentrations 0, 1, 5, 50, 100, 200, 300, 400 and 500 ppm of both gases. The working temperatures were 23 (ambient), 150, 200, 250 and 300 °C. The tests were performed in three steps: (1) the pellets were heated in air at the cited temperatures and were let to rest for 5 min for their thermal stabilization; (2) at every temperature, the CO and the C_3_H_8_ were allowed to flow, recording the variation of electric resistance; (3) the sensitivity changes (S) were evaluated using the equation [15,29,30,31]:

(1) $$S=\frac{G_{CO/propane}-G_{air}}{G_{air}}$$

where, *G_CO/propane_* and *G_air_* are the pellets’ conductance (1/electric resistance) in the test gases.

**Figure 7:**
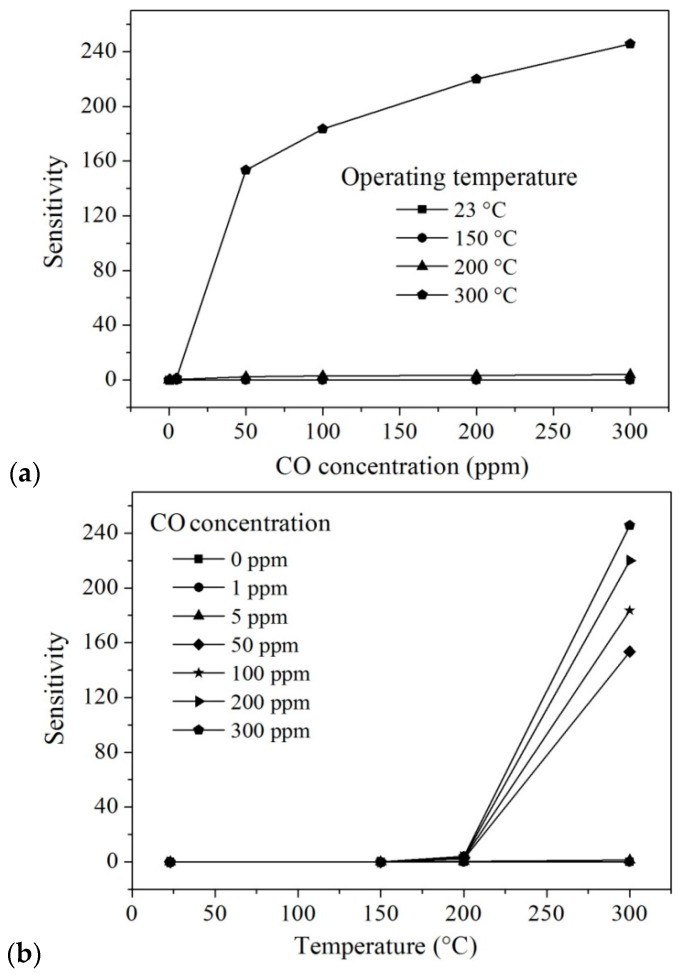
Sensitivity of MgSb_2_O_6_ pellets: (**a**) S *vs.* CO concentration; (**b**) S *vs.* operating temperature.

Figure 7a,b show the sensitivity tests; Table 1 summarizes the sensitivity variations in carbon monoxide (CO). According to these results, the MgSb_2_O_6_ nanorods are highly sensitive to concentrations of CO at the given operation temperatures. However, at temperatures below 150 °C no sensitivity changes were detected. As expected, the maximum sensitivity values were at the maximum CO concentration at the given temperatures; a sensitivity of ~245.75 in a CO atmosphere at 300 °C for a gas concentration of 300 ppm. When the carbon monoxide made contact with the MgSb_2_O_6_ pellets at moderate temperatures, the adsorbed CO reacted with the oxygen anions chemisorbed on the surface, yielding CO_2_ and a release of electrons back into the conduction band [18,32]. A possible reaction between the CO and similar materials to one used in this work has been discussed in previous works [18,32,33].

**Table 1:** Sensitivity values of MgSb_2_O_6_ in CO atmospheres.

Temperature (°C)	Concentration CO (ppm)	Sensitivity (S)	Temperature (°C)	Concentration CO (ppm)	Sensitivity (S)
200	0	0	300	0	0
	5	0.36		5	1.37
	50	2.40		50	153.47
	100	2.84		100	183.46
	200	3.39		200	219.93
	300	3.87		300	245.75

In general, the gas-sensing mechanism of materials like the MgSb_2_O_6_ is based on the change of the electrical resistance or conductance produced by electron transfer due to the adsorption and desorption of oxygen over the MgSb_2_O_6_ pellets [34,35,36]. Below 150 °C, the available oxygen species are mainly $O_{2}^{-}$, while at temperatures above that the more reactive species $O^{-}$ and $O^{2-}$, are predominant [37,38]. At 150 °C, the thermal energy is not enough to provoke the oxygen’s desorption reactions, meaning that no electrical signal could be detected, no matter the gas concentration [15,18]; conversely, at a higher temperature (like at the tested temperatures of 200 and 300 °C) more oxygen species are generated, provoking a rise in the CO-solid interaction [39] and consequently an increase of the sensitivity [18].

**Figure 8:**
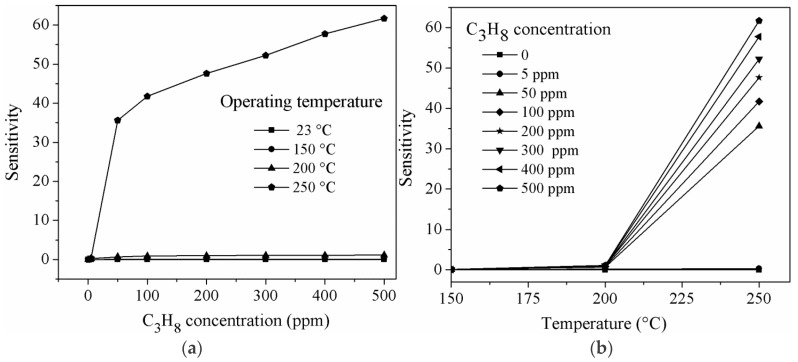
Sensitivity of MgSb_2_O_6_ pellets: (**a**) C_3_H_8_ concentration; (**b**) operating temperature.

Sensitivity to propane (C_3_H_8_), as a function of the gas concentration at the given temperature, is depicted in Figure 8a,b; Table 2 summarizes the sensitivity results for such gas.

**Table 2:** Sensitivity values of MgSb_2_O_6_ in C_3_H_8_ atmospheres.

Temperature (°C)	Concentration C_3_H_8_ (ppm)	Sensitivity (S)
250	5	0.311
	50	35.62
	100	41.72
	200	47.62
	300	52.20
	400	57.75
	500	61.66

As for CO, the nanorods show high sensitivity to propane concentrations and operation temperatures. However, at temperatures 23 (ambient) and 150 °C, no response was detected. Conversely, when the temperature increased to 250 °C, the sensitivity rose to the maximum (S ~ 61.66) at the highest concentration (500 ppm). The sensitivities were roughly: 0.311, 35.62, 41.72, 47.62, 52.20, 57.75 and 61.66 for the concentrations: 5, 50, 100, 200, 300, 400 and 500 ppm, respectively. The observed sensitivity trend has been widely reported in the literature [40]. This sensitivity rise can be attributed to the oxygen desorption that occurs at temperatures higher than 150 °C [29], which is probably due to the interaction of the propane molecules with the material’s surface when the temperature increases [30] (in our case, at 250 °C). Also, the gas sensitivity depends on the temperature and involves the chemisorption of the oxygen and its subsequent reaction with the sampled gas [29,30,41,42]. Therefore, at a given temperature, the sensitivity depends on the oxygen’s partial pressure and the adsorption-desorption kinetics [41,43]. Notwithstanding that results are not shown here for when the gas chamber was evacuated, the material’s sensitivity went back to its baseline, guaranteeing that the material could be reused. Regardless, the excellent results shown here assure that this material can be perfectly used at least once.

According to Figure 8, the propane detection mechanism at 250 °C is not quite obvious. Some authors have studied the catalytic and detection properties of metallic oxides, like the one studied here, for propane, and have proposed some mechanisms involving a relative low kinetics in the presence of oxygen, without oxygen, and mixtures of propane with other gases [42,43,44,45,46,47].

The propane sensitivity results were compared with similar previous works [29,30], finding that we have succeeded obtaining a better sensitivity to such gas. For example, in references [15,29,30], it is stated that: (a) LaCoO_3_ showed a maximum sensitivity of ~42 at a temperature of 350 °C and a C_3_H_8_ concentration of 300 ppm; (b) a sensitivity of ~0.7 and ~0.6 was reached for SnO_2_ at 300 °C and a propane concentration of 500 ppm; (c) for ZnO, the maximum sensitivities were 2.25, 3.6 and 5.8 at 300 °C and a gas concentration of 300 ppm. We have recently reported that a maximum sensitivity of 4.8 was reached for CoSb_2_O_6_ at 350 °C and a C_3_H_8_ concentration of 300 ppm [18]. It is therefore important to emphasize that a sensitivity of ~62 at 250 °C and a propane concentration of 500 ppm was obtained in this work. Part of this success is due to the fact that the gas detection ability of a semiconductor material depends on the morphology and the particle size [48]. When the particle size is fine enough (in our case, of nanometric size), the sensitivity increases considerably [7,18,49,50,51,52]. In addition, the smaller structures (“almost 1D”) show the higher thermal stability and the better electrical conduction [34,42]. All these advantages have been verified during this work.

## 4. Conclusions

The colloidal method is a convenient synthesis method (as an economically cheap process, compared with alternatives) for the preparation of MgSb_2_O_6_ nanorods, because it is possible to have greater morphology control for the final structures. The MgSb_2_O_6_ nanorods are clearly sensitive to the tested gases at temperatures above 150 °C. A uniform response to the operating temperatures and gas concentrations was obtained in carbon monoxide (CO) and propane (C_3_H_8_) atmospheres. The maximum sensitivity was ~245.75 in a CO atmosphere at 300 °C for a gas concentration of 300 ppm. The high sensitivity of the material is attributed to the nanometric-sized structures obtained during the synthesis process. MgSb_2_O_6_ oxide is therefore very suitable for use as a gas sensor.
